# The female presence in different organisational positions and performance in secondary schools: Does a woman leader function as mediator?

**DOI:** 10.1371/journal.pone.0222411

**Published:** 2019-09-26

**Authors:** Irene Campos-García, José Ángel Zúñiga-Vicente

**Affiliations:** Rey Juan Carlos University, Faculty of Legal and Social Sciences, Business Administration (ADO), Applied Economics II and Foundations of Economic Analysis, Paseo de los Artilleros, Madrid (SPAIN); Middlesex University, UNITED KINGDOM

## Abstract

This study examines the relationship between female representation in different organisational positions and performance using a sample of Spanish secondary schools. These organizations have been usually depicted as ‘feminised environments’ although women in managerial positions are still underrepresented. Based on different theoretical approaches, we separately investigate the relationship between a greater female presence and school performance in three positions: a) as principal; b) on the management team; and c) as teachers. We also investigate if having a female leader exerts a significant mediator role on the relationship between greater female representation on the management team and teachers, respectively, and school performance. Our results reveal a positive and significant relationship between having a woman principal or a greater proportion of women teachers and school performance. However, the relationship between a high proportion of women on the management team and school performance is negative. We also find that a female principal does not play a significant role as mediator in the relationship between having a greater proportion of women on the management team and as teachers and school performance.

## Introduction

During the last few decades, women have achieved greater representation in the labour market. In 2018, the rate of female participation in the global labour force was 48.5% compared to the 75% male rate. Although women’s participation rates are gradually approaching those of men, mainly in developed and emerging countries, there is still a gender gap in labour force participation—i.e. the difference between male and female participation rates. Overall, this gender gap stood at 27% in 2018, compared with 29.1% in 1990 [1]. Yet this gender gap is even greater when it comes to the global representation of women in leadership positions. Women held under a quarter (24%) of senior managerial roles across the world in 2018 [2]. They are still underrepresented as directors or in the position of maximum responsibility. Actually, the number of women leading organisations (whether from the social, political, or business sphere) has always been relatively small. For example, there are currently 29 female heads of state and government and just 24 female CEOs among the 2018 Fortune Global 500 companies [3], [4].

Certainly, this gender imbalance has placed women at the centre of the political and social debate in most countries [5], [6], [7]. Both academic literature and the media have also paid increasing attention to women, leading to terms such as ‘glass ceiling’, ‘glass cliff’, ‘glass labyrinth’, ‘glass walls’, and ‘sticky floor’ to refer to the impediments or barriers women usually face as they attempt to climb the organisational ladder [8], [9]. In fact, achieving greater female representation in the labour market and, especially, in managerial positions is now an issue of active policymaking in many countries around the world, with some governments establishing the need for specific female quotas in organisations, while others provide sundry guidelines for achieving greater female representation through the launch of equality programmes (e.g., [10], [11], [12]). Ultimately, as noted by Frink et al. embracing and supporting greater female representation is being hailed by policymakers in most sectors of employment—public and private, for-profit, and not-for-profit—and by practitioners and scholars alike as a core value that signals the right way to proceed in organisations, implying that it may lead to higher organisational performance [13].

In recent years, scholars have been especially concerned with examining the relationship between a greater female presence and performance. There are several theoretical approaches from different fields of study (e.g., economics, management, organisational behaviour, and social psychology) that provide contradictory arguments regarding this relationship. This might explain why the extant empirical evidence is mixed. Some researchers have also suggested that this relationship might be, at least in part, dependent on the specific organisational context in which men and women work (e.g., [13], [14], [15], [16], [17]). In this paper, we focus our attention on educational organisations (secondary schools) that have been usually depicted as ‘feminised environments’, as women represent the majority of the workforce, although they tend to be still underrepresented in managerial positions in most countries around the world [18], [19], [20].

The purpose of this study is twofold. First, we examine the relationship between the female representation in different hierarchical positions and performance. Specifically, we separately investigate the relationship between a greater female presence and performance in three positions: a) as principal; b) on the management team; and c) as teachers. Second, we investigate the potential mediator role of having a female principal on the relationship between greater female representation on the management team and as teachers, respectively, and performance. The empirical analysis is conducted on a sample of Spanish secondary or high schools.

This study contributes to the literature in the following ways: On the one hand, most empirical studies so far—in both the business and educational fields—have focused on separately exploring the effect that the presence of women in different positions may have on performance (e.g., [13], [15], [21], [22], [23], [24]). To our knowledge, however, there are virtually no studies that have simultaneously examined the direct and mediating effects of women in different executive and non-executive positions in organisations, as well as the latter’s performance. This study therefore provides a more complete and realistic snapshot of the female representation–performance link, as it will allow us to discover in which roles greater female presence may be more beneficial for organisations in terms of performance.

On the other hand, the evidence shows that there are very different proportions of women and men in the workforce and managerial positions across sectors. For example, according to the report *Progress of the World’s Women 2015–2016*, the presence of women in the overall workforce is predominant in the services sector, especially in education, health, and social work [25]. Compared to most business sectors, in education there is also observed a greater presence of female administrators (e.g., [26], [27]). In fact, this study also echoes the need to control the organisational context in which the female representation–performance link is examined. There are still few studies considering this circumstance (e.g., [13], [14], [23], [28]). As argued in these studies, the consideration of a homogeneous organisational context in the analysis is interesting because it might help explain certain inconsistencies in past research and provide a more precise understanding of the female representation–performance link. It is therefore obvious that more empirical research is required to clarify this link, and this study is one step further in this direction. In addition, knowing how women, depending on the positions they occupy in the flow chart, impact the performance of educational organisations may be especially important to help improve the success of a country’s educational system.

## Model and hypotheses

**Fig 1:**
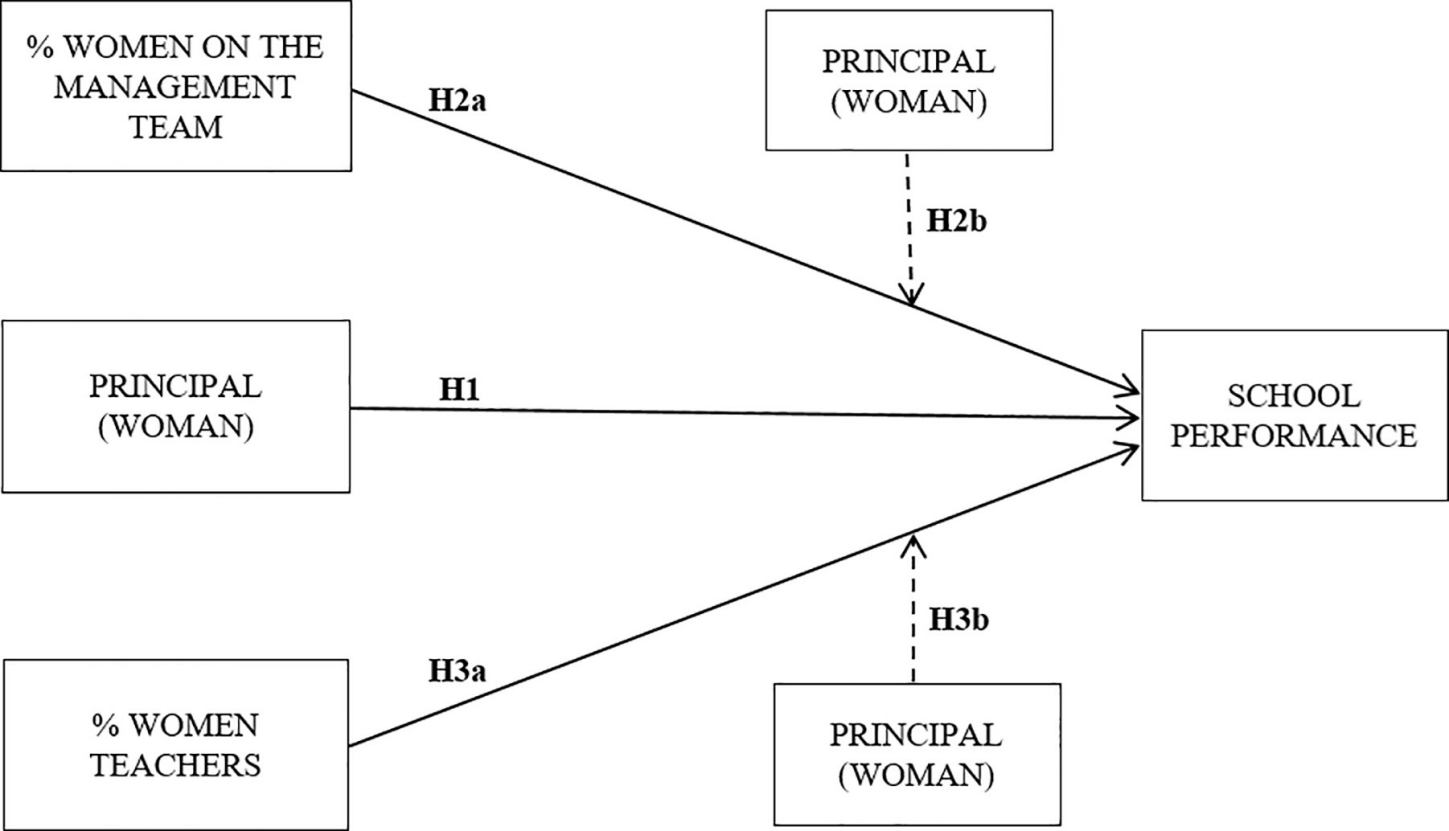
Relationships between the female presence in different hierarchical positions and performance.

Fig 1 summarises the proposed model. We assume that female representation has a relationship with performance in secondary schools. In this regard, we examine the type of individual relationship between the female presence and performance in three hierarchical positions: as principal (Hypothesis 1), on the management team (Hypothesis 2a), and as teachers (Hypothesis 3a). Additionally, we investigate whether the relationship between women on the management team and as teachers and performance may be conditioned (mediated) by the fact that the school is also led by a woman (Hypotheses 2b and 3b, respectively) (see Fig 1).

### Women as organisation leaders

An extensive academic literature on gender stereotypes and leadership characteristics has been developed in recent decades. Prior literature based on *Social Role Theory* reveals that the potential disparities found in the leadership behaviours of women and men are governed by differences in their roles (e.g., [29], [30], [31]). Specifically, according to stereotypic beliefs about the sexes propounded by this theory, women are viewed as more communal (selfless and concerned with others) and less agentic (self-assertive and motivated to dominate) than men [30]. On the other hand, men tend to be viewed as more effective in the roles and traits considered more masculine—these include, for example, being more aggressive, competitive, independent, or self-confident. Proponents of this theory attribute the persistence of such behavioural patterns from generation to generation to cultural processes (e.g., [29], [32]). In turn, other researchers (e.g., [31]) argue that the origins of sex differences in social behaviour lie mainly in evolved dispositions that differ by sex or in the differing placement of women and men in the social structure.

The past years have witnessed the emergence of a large body of research interested in empirically exploring the relationship between the presence of a female leader and performance. In a recent meta-analysis of 146 studies conducted in 33 different countries, Jeong and Harrison show that the relationship between leader gender and long-term organisational performance is statistically significant but tiny [33]. Actually, most studies suggest that the existence of a positive relationship between a female leader and performance is precisely due to differences in the social behaviours of women and men when they make strategic decisions (e.g., [34], [35], [36], [37]).

Though educational organisations have widely been described as ‘feminised’ work environments, the barriers to management level and the underrepresentation of women in school principalship is well known—on average in developed countries, just over one third of principals are women [38]. Overall, the proportion of women leaders has usually been greater at lower educational levels and in smaller schools [26], [27], although this situation is changing somewhat in recent years as the presence of female principals is also increasing in higher education levels in several countries (e.g., [39], [40]). As in other types of organisations, this is obviously due to a variety of obstacles or barriers hindering the access of female teachers to leadership positions. In line with the above arguments about the origins of sex differences in social behaviour, studies argue that such obstacles can be internal in nature (differences in education or the fear of not living up to the expectations of the female role) and external (co-optation or the existence of a male-dominated organisational culture) (e.g., [20], [41], [42]).

Past research has shown that gender-specific behaviours affect school principals’ practices and, thus, may influence the individual performance of students as well as the overall performance of schools [43]. More specifically, based on a meta-analysis, Hallinger, Donguy, and Wang find statistically significant gender differences in instructional leadership adopted by principals, with female principals engaged in more active instructional leadership than their male counterparts [44]. In this regard, some studies emphasise the potential benefits associated with female leader values and behaviours in schools (e.g., [45], [46], [47], [48], [49], [50], [51]). For example, Hall finds that, as a result of such values and behaviours, it is more likely that school decisions made by a female principal are based on the best interests of students and subordinates, not necessarily on power [46]. In a similar vein, Trinidad and Normore and Williamson and Hudson point out that women usually value close relationships with students, staff, colleagues, parents, and other stakeholders as a key aspect of school leadership when making decisions and solving problems [50], [51]. As also suggested by Trinidad and Normore, it is precisely women’s greater concern for shared decision-making and problem-solving with all the school’s stakeholders that leaves enough space to deviate from the more hierarchical or autocratic systems of approval and to concentrate on the search for the best solution to most problems/issues in the interests of the greater good [50]. Fennell also concludes that women principals tend to view power as an enabling and positive energy for change and growth in schools rather than a source of ‘top‐down’ domination [45]. On the other hand, some studies confirm that teachers tend to be more involved, collegial, and cooperative with administration (e.g., [52]), and exemplary and recognised state ratings (e.g., [53]) or school students’ outcomes (e.g., [21]) can significantly improve when the principal is a woman. Accordingly, a positive association between a female principal and school performance is expected. This leads us to predict:

*Hypothesis 1*: The fact that a secondary school is led by a woman will have a positive relationship with its performance.

### Women on the management team

It is true that the number of women on boards of directors or in top management positions has increased in most sectors and countries in the last 20 years. However, women are still underrepresented at the highest levels of the corporate hierarchy, as noted in recent reports (e.g., [54], [55]). In fact, it is observed that in organisations of most countries examined, the proportion of women on boards/management teams is still far from 50%. Across a list of 46 developed and emerging countries, the Deloitte report highlights that there is effectively considerable cross-country variation in female board representation, with some of the lowest rates in South Korea (2.5%), Japan (4.1%), Morocco (4.3%), the Russian Federation (5.8%), Mexico (6.0%), and Chile (6.5%), and some of the highest rates in Norway and France (40%), Sweden (31.7%), Italy (28.1%), Belgium (27.6%), and New Zealand (27.5%). In most countries examined, this percentage ranges between 10% and about 20%: Peru (10%), China and Singapore (10.7%), Turkey (11.5%), the United States (14.2%), Switzerland (14.8%), Spain (16.3%), Ireland (16.5%), the United Kingdom (20.3%) [55]. These reports, together with other studies of a descriptive nature, examine the evolution of women in different managerial positions, although this numerical account provides an incomplete analysis of the value attributed to women in the upper hierarchy of an organisation [56], and it sometimes reflects no more than a token nod to the obligation of reaching specific gender quotas [57], [58].

Theoretically, the *Value in Diversity* and *Information/Decision-Making Perspectives* might help to understand why greater female representation on the management team may be positively related to organisational performance, especially, as is the case, when female representation in this position is still low. For example, according to the proponents of the first view, greater female presence should provide a greater range of perspectives, belief systems, and potential internal and external networks to more effectively address the problems or tasks at hand (e.g., [59], [60], [61], [62]). Similarly, the defenders of the second view also suggest that greater female representation is more likely to be related to a broader range of task-relevant knowledge, skills, and abilities, and may lead to more creative and innovative ideas and solutions (e.g., [63], [64]). All this may be especially relevant in management team’s decision-making processes, as they are usually involved in non-routine problems or task solving. Accordingly, a greater proportion of women may bring added knowledge and fresh perspectives on complex issues, which can help correct informational biases in formulating strategies and solving problems (e.g., [15], [59], [65]).

Other studies also recognise that women can add important symbolic value both inside and outside the organisation, and greater female representation on management teams can substantially enhance their legitimacy or reputation from a social perspective (e.g., [66], [67], [68], [69]). Additionally, some studies stress that women seem to be more cooperative than men and may therefore contribute to creating a more cohesive working environment on the management team, which may be useful for addressing and solving complex problems (e.g., [70], [71]). In this regard, several studies report that women are more cooperative than men in social dilemmas [72], more altruistic than men [73], [74], more honest than men in the die-under-cup paradigm [75], more averse than men in personal moral dilemmas [76] and also tend to exhibit a weaker inclination to enter competition [77], [78].

The study of the linkage between female representation on boards/management teams and performance has also been the subject of a lively debate in recent years. Three meta-analyses summarise the main results. Pletzer et al., based on 20 prior studies, find that the average correlation between the proportion of women on the board and performance was small and not statistically significant [16]. However, Post and Byron, combining the results from 140 studies, show that female board/management team representation is positively related to firm performance—i.e. firms with a greater proportion of female directors tend to have slightly higher accounting returns than firms with fewer female directors. This relationship is statistically significant, suggesting it is not a ‘chance effect’ [17]. Based on a sample of 87 studies, Byron and Post also find a positive female board/management team representation–social performance linkage [79].

Although the proportion of women on the management teams in the educational sector is greater than in other business sectors such as materials, energy, information technology, and industry [80], women are still underrepresented. In general, the main role of the management team is to manage the school with a view to providing the best possible education and educational opportunities for all of its students. Among other functions, this involves, for example, setting the strategic orientation for the school and its education project, encouraging the participation of the educational community, and managing the human and material resources of the school. As a general rule (e.g., in Spain), the management team has between three and six members, depending on the size of the high school.

To a certain extent, this greater proportion of women might also be explained by the fact that a significant degree of female values and roles is frequently required in the educational area, which is also in consonance with the arguments behind *Social Role Theory*. Many women have been appointed as administrators precisely for their caring and sharing attributes [81]. In this vein, Limerick and Lingard recognise women’s special contribution to school managerial positions as communicators, facilitators, and collaborative managers [82]. Moreover, as suggested by Krüger, “because they are different, men and women working together have more alternative strategies at their disposal than either acting alone […]. A mix of masculine and feminine elements in school management leads to a broader repertoire of behavior and consequently to more flexible action” [47].

On the other hand, the aspects of enhanced decision making, the depth of consumer (students) insight, and strengthened employee (teacher) engagement that are frequently outlined in the business world when there is greater female representation in management teams can also be applied in the context of schools. Because women are still underrepresented on management teams of most secondary schools, then it seems reasonable to assume that a greater proportion of women on management teams may mean more robust debate, better decision making, and improved educational outcomes for schools. This leads us to predict:

*Hypothesis 2a*: There will be a positive relationship between a greater proportion of women on the management team and performance in secondary schools.

In addition, we argue in favour of a potential mediator role of a female principal on the relationship between the proportion of women on the management team and performance. Importantly, the evidence shows that organisations with a woman in the top leadership position have almost twice as many women in the boardroom as organisations led by a man [55], [83]. Several arguments are invoked to explain this difference in management team composition between organisations with female versus male leaders [83]: On the one hand, the appointment of a woman as leader might mean the removal of an institutional barrier against women ascending to top leadership positions, so it becomes easier for other women to reach managerial positions. On the other hand, the manager applicant pool may be more diverse, so when a seat on the board/management team becomes available, these organisations may be in a better situation to exploit the leader’s connections. Finally, the presence of a female leader may indicate that an organisation’s culture is also more amenable to female managers in general. These organisations are also more likely to appoint female managers and a new female leader in the future.

According to *Social Role Theory*, as the proportion of women on the management team increases, and the leader is also a woman, several female stereotypes are increasingly likely to be applied to the men as well (e.g., [84]). As noted above, this may be especially expedient in educational organisations (especially in primary and secondary schools), as a significant degree of female values and roles is frequently required. In a context like this, it is also more likely that personnel relationships matter more than status and power, and information is shared more easily as women tend to manifest a high degree of empathy, engagement, and involvement with their teams, and hence a greater ability to engage with others [85].

On the other hand, according to the *Similarity-Attraction Approach*, to minimise uncertainty at work, women principals are more likely to sponsor the mobility of others who have demographic characteristics like themselves [58]. This greater similarity in gender in different hierarchical positions is supposed to make communication easier, encourage relationships of trust and reciprocity and, ultimately, improve organisational effectiveness and performance [86], [87]. Moreover, in a context like this, women principals are also expected to contribute to advancing the careers of other women teachers. In this regard, some studies have found that more women teachers are sponsored to hold managerial/administrative positions under women principals (e.g., [86], [88]). More specifically, it is highlighted that the ‘feminine’ leadership style, emphasising caring, empowerment of others, and less hierarchical/authoritative relations [86], [89], [90], [91], can provide opportunities for relations of sponsorship that advance women’s professional careers within the school, which might have an impact on school performance. Thus, we state:

*Hypothesis 2b*: The presence of a female principal will mediate the relationship between a greater proportion of women on the management team and performance in secondary schools.

### Women in the workforce

As noted above, the number of women in the workforce has increased in the past few decades in many countries all over the world, mainly in developed and developing countries. However, on average, since 1990 the gender gap in world labour force participation has narrowed by just 2.1%, with the bulk of the reduction occurring in the years up to 2009. It is true that women’s participation rates in the labour force are gradually approaching those of men in many developing and developed countries. On average, this gender gap has reduced by 11.8% and 15.6% in 2018, respectively, and the gender gap in participation rates in these group countries is the lowest recorded since 1990. However, in the group of developed countries, it remains wide in several countries, especially in Southern Europe. In the group of emerging countries, the gender gap is 30.5% [1].

The use of work teams consisting of an increasing proportion of women has become common practice in most modern organisations of many countries. Based on the *Value in Diversity Perspective*, several researchers have traditionally argued in favour of a greater proportion of women in the workforce (especially when this proportion is low) on the assumption that this should have a positive effect on organisational performance (e.g., [13], [92]). Overall, as also noted above, proponents of this approach argue that greater female representation may enrich the workplace by broadening employee perspectives, strengthening their work teams, and providing greater resources for solving complex problems [93], [94], [95], [96], [97].

The empirical study by Frink et al. is a pioneer work in exploring the nature of the link between the proportion of women in the workforce and organisational performance [13]. This study highlights the importance of the specific context in which the relationship is explored. In fact, these authors recognise that the potential benefits of greater female representation may vary significantly with work tasks or situations and are more likely to be attained in those tasks or situations based less on physical attributes (e.g., service/wholesale/retail sectors). Other studies conducted in different organisational contexts have corroborated the existence of a positive relationship between greater female presence in the workforce and performance (e.g., [95], [98], [99] [100], [101]).

As noted above, education is one of the sectors with a greater proportion of women in the labour force. Specifically, the female presence as teachers prevails in most countries at initial educational levels (i.e. early childhood and primary education); it is also high at intermediate levels (i.e. secondary education) and diminishes significantly at the highest levels (i.e. university or tertiary education). For example, in 2015 (the year in which we collected data for our study) the percentage of female teachers in secondary education was as follows: 65.13% in Austria, 62.94% in Belgium, 64.81% in Brazil, 59.91% in Chile, 51.91% in China, 62.37% in Germany, 59.44% in Greece, 43.15% in India, 70.87% in Italy, 52.07% in the Netherlands, 62.67% in New Zealand, 62.65% in Norway, 69.27% in Poland, 59.19% in the Republic of Korea, 56.93% in Spain, 63.92% in Sweden, 62.01% in the USA, and 62.85% in the UK [102].

All in all, it is important to note that outcomes in secondary schools (especially students’ academic achievements) may be heavily dependent on teachers. In terms of teachers’ impact on academic performance, several studies have sought to identify those active teaching effects (related to teachers’ interpersonal behaviour) and passive ones (teachers’ race, ethnicity, or gender) that help to increase teacher effectiveness (e.g., [103], [104], [105]). Specifically, there is empirical evidence confirming that the pattern of teacher–student interactions may be related to a teacher’s gender, since male and female teachers can significantly differ in terms of their classroom management practices and behaviours (e.g., [106], [107]). Regardless of their gender, male and female teachers can be equally effective, good teachers, and equally respected and admired among students. Nevertheless, given the specific context, and precisely as a result of the potential linkage between female teachers’ abilities and traits and students’ behavioural and emotional needs in secondary education—favoured by physical, cognitive, and psychological changes—and in line with prior studies (e.g., [22], [24], [108], [109], [110]), we suggest that a greater proportion of female teachers might have a positive impact on school performance. This is also more likely to occur because, according to the *Similarity-Attraction Approach*, more homogeneous work groups in terms of, for example, teachers’ gender could encourage better communication, stronger cohesion, and lower conflict within the school [86]. Thus, we state:

*Hypothesis 3a*: There will be a positive relationship between a greater proportion of women teachers and performance in secondary schools.

In addition, we also suggest a potential mediator role of a female principal on the link between a greater proportion of women as teachers and performance. In exploring the relationship between superior–subordinates and performance, most studies have relied on the *Relational Demography Perspective* (e.g., [111], [112], [113], [114]). This view suggests that demographic similarities in superior–subordinate relations invoke a dynamic attraction, whereby demographically similar individuals accentuate each other’s positive attributes and derive a positive social identity [114]. In this sense, Tsui and O'Reilly argue that the increasing dissimilarity in certain superior–subordinate demographic attributes is related to a lower effectiveness perceived by superiors, less personal attraction for subordinates among their superiors, and increased role ambiguity experienced by subordinates [113].

In terms of gender, numerous studies claim that it may be an important social category to explain the differences in extra-role behaviour and task performance. For example, Green, Anderson, and Shivers find that gender differences have a significant effect on leader–subordinate exchanges, showing that such exchanges are of lower quality when the leader and subordinates are of different genders [115]. Pelled also posits that gender dissimilarity may increase the perception of emotional conflict, indirectly affecting members’ confidence in their group [111]. In turn, Tsui and O'Reilly show that the subordinates in mixed-gender dyads were rated as performing more poorly, and they reported higher levels of role ambiguity and role conflict [113]. These authors also highlight that woman subordinates with woman superiors reported the lowest level of role ambiguity, were rated to be most effective, and were more liked by their superiors. Eagly and Carli also reveal that women leaders achieve the best organisational performance in terms of effectiveness, satisfaction, and the extra effort of their subordinates when women also make up a significant proportion of the labour force [116]. This may also be in keeping with the notion that subordinate women can increase their confidence and motivation through the woman leader’s empowerment [117].

In the educational field, some authors show that under female principals, both male and female teachers express more positive attitudes toward their workplace than under male principals [118]. In a similar vein, Lee et al. find that female teachers feel more empowered when working in schools headed by female principals [88]. They underline that certain measures of female teacher power as self-efficacy, locus of control, and staff influence over policy are higher when working for female than for male principals. Likewise, Ballou and Podgursky reveal that teachers tend to rate a principal of their own sex (or race) higher, although this effect is more pronounced for female than male teachers [119]. Moreover, Husain, Matsa, and Miller explore how female principals affect rates of teacher turnover—usually viewed as an important determinant of school quality—and find that male teachers are more likely to voluntarily leave their schools when they work under female principals, while such effects do not exist for female teachers [120]. The findings of most of these studies are also consistent with the arguments from the *Similarity-Attraction Approach*, as women teachers may significantly benefit from working under the supervision of other women principals, and this circumstance may have consequences for the school (e.g., [86]). Therefore, we hypothesise that a female principal may mediate the relationship between women teachers and school performance:

*Hypothesis 3b*: The presence of a female principal will mediate the relationship between a greater proportion of women teachers and performance in secondary schools.

## Method

### Sample and data collection

The empirical analysis for testing our hypotheses is based on data from a survey of Spanish secondary schools. According to the latest available data, women represent 36% of school principals in Spanish secondary schools, 49% of studies coordinators (i.e. management team), and about 57% of teachers [102], [121], [122], [123]. The figures on the presence of women in the principalship and school administration are similar in many countries around the world; however, the percentage of women teachers is lower than the average of the OECD countries, and Spain stands at the bottom of the ranking in terms of female teaching staff [102], [122], [124].

The survey was administered in the Community of Madrid. It collected information on different issues related to the schools’ organisation and operation, their management teams, and teachers. We drafted many of the questions in the survey following the guidelines of the OECD Teaching and Learning International Survey (TALIS)—Principal Questionnaire.

The first step in the sampling process involved identifying the total population of secondary schools in the Community of Madrid. We retrieved the information available on the website of the Department of Education of the Community of Madrid. According to this information, the target population consisted of 595 schools (the total number of secondary schools and, hence, principals in the Community of Madrid when the study was conducted). The second step involved the preparation of a questionnaire that was emailed to the principal of each school. Although many of the questions included in our questionnaire were based on the TALIS guidelines, the questionnaire was first reviewed and discussed by several academics. Specifically, the questionnaire was reviewed and discussed with four colleagues (two men and two women) from three Spanish universities who were familiarised with different aspects related to gender and/or top management teams. Additionally, we held face-to-face interviews with the principals and several teachers from two schools in order to receive feedback on the clarity of the questions included in the questionnaire, thereby ensuring that unfamiliar and ambiguous terms or issues were not included in any of the questions and that the questionnaire was as concise as possible.

A customised survey was considered the most appropriate manner in which to collect data because, to our knowledge, detailed archival information on the issues examined was not available from secondary sources. Data were collected between May and September 2015. After three follow-up reminders, a total of 105 usable questionnaires were returned via email, which represents about 17.65% of the target population. This response rate is comparable to some previous studies using this type of primary source (e.g., [125]).

We performed a *χ*^*2*^ test and *one-way ANOVA* to check whether there were significant differences between the reference population and our study sample. The variable ‘district’ was used for conducting the *χ*^*2*^ test, as secondary schools are grouped by districts or geographical areas in the Community of Madrid (Madrid City, Madrid North, Madrid South, Madrid West, and Madrid East), whereas the main dependent variable of interest (i.e. *school students’ achievement*) was used for conducting the *one-way ANOVA*. The *χ*^*2*^ and *F* values were 6.653 (*p =* 0.155) and 0.496 (*p =* 0.482), respectively. Thus, there were no statistically significant differences between the schools included in the whole population and the final sample in terms of geographical areas and performance. For the performance variable we also conducted two-sample *t* tests because we had complete information on it on the website of the Department of Education of the Community of Madrid. Our results reveal that there are no statistically significant differences between the mean values of *school students’ achievement* of our sample and the remaining schools that were not included in our study (*t =* 0.704; *p =* 0.482). To a certain extent, all these findings could be interpreted as a clear indication of sample representativeness and the potential absence of selection bias in our empirical study.

### Variable measurements

The dependent variable of interest in this study is *school students’ achievement*. One of the main indicators of performance linked to schools involves the outcomes achieved by their students. In this regard, it is important to point out that students’ academic outcomes have traditionally been considered a good proxy for assessing a school’s success/effectiveness (e.g., [126], [127], [128]) and, hence, also to assess the potential influence that the principal and teachers may have on school students’ achievement (e.g., [21], [24], [110]). This variable is computed as the average score obtained by each school in the university admissions test performed at the end of the academic year, and it is similar to other students’ achievement measures used by prior research examining school success/effectiveness (e.g., [110], [126], [129], [130]). It is a continuous variable with values between 0 and 10. In Spain, this is the only objective way of comparing the students’ achievement across different high schools because all students sit for the same test. The decision to use this measure is also based on the premise that this variable provides a valid measure of students’ secondary school learning and achievement. Moreover, it is usually argued that other measures (e.g., cumulative or overall grade point averages) can be more subjective due to the varying standards and purposes teachers associate with grades, whereby grading practices can significantly vary across schools, contributing to the phenomenon referred to as grade inflation (e.g., [129], [130], [131]). The information on this variable was obtained from the website of the Department of Education of the Community of Madrid.

The main independent variables used to test the subsequent hypotheses were built from the information obtained from the questionnaire directly administered to the principals, as follows: *female principal*, *women on management team*, and *women teachers*. The former is a dummy variable that takes the value of 1 when the principal is a woman, and 0 otherwise (i.e. the principal is a man). The second and third variables are calculated as the proportion of women on the management team and in the overall workforce (i.e. as teachers and where the members of the management team are excluded from the calculations of this variable), respectively. This has been a common way of assessing the influence of women on performance in both the business and educational sectors (e.g., [13], [21], [23], [110]).

A set of control variables, whose effects have been reported in prior research, especially in the field of educational organisations, is also included in the empirical study (e.g., [21], [110], [132]). We also added these control variables to minimise the risk of omitted variable bias. We control for the potential effect of *school ownership* by considering a variable that takes a value of 1 if the school is public, and 0 otherwise (i.e. it is privately owned). The information to build this variable was obtained from each school’s website. The remaining control variables were obtained by directly asking principals. *School size* is measured by the total number of secondary-level teachers; *foreign students* is operationalised as the proportion of foreign students over the total number of students; *teacher stability* measures the proportion of teachers with more than five years’ service in the school (as the five years of seniority in employment are usually considered as a good indicator to measure the percentage of novel or veteran teachers [133]); and *teacher motivation* is defined as the percentage of motivated teachers over the total number of secondary-level teachers at school. Based on the Watt and Richardson classification—highly engaged persisters, highly engaged switchers, and lower engaged desisters—principals were also asked to indicate the percentage of teachers for each group in their respective organisations [134]. This is a fairly appropriate way to measure employee motivation because the school principal is in daily personal contact with his/her staff and can thereby see which teachers effectively have a greater level of motivation and engagement at work. Here, we also underscore the usefulness of questioning someone on someone else’s behaviour (i.e. principals) and who is close to or knows the target individual quite well [135], [136]. Thus, *teacher motivation* is defined as the percentage of teachers who showed motivation, a positive attitude, and commitment to his/her school over the total number of teachers.

### Method of statistical analysis

Hierarchical multiple regression analysis is used to test the hypotheses regarding the relationship between the independent variables of interest and the variable representing performance (i.e. *school students’ achievement*). First, the control variables were regressed on the dependent variable (this is the basic model, or Model 1). Second, the three independent variables of interest were separately regressed on the dependent variable (Models 2–4). To test *Hypotheses 2b* and *3b*, which predict the mediator role of a female principal on the relationship between women on the management team and as teachers and performance, we adhered to the procedure outlined by Baron and Kenny [137] and Judd and Kenny [138] and that has also been used by other researchers in studies on gender (e.g., [139]). First, the independent variable of interest must influence the dependent variable (Models 2–4). Second, the independent variable of interest must also influence the presumed mediator. Because the mediator variable (i.e. *female principal*) is dichotomous, here we estimated a logistic regression. Third, the mediator must also influence the dependent variable while controlling for the independent variable of interest (Models 5 and 6, respectively). Finally, a previously significant relationship between the independent and dependent variables must be significantly reduced in the presence of the mediator variable (Models 5 and 6). According to Baron and Kenny “Perfect mediation holds if the independent variable has no effect when the mediator is controlled” [137].

We also provide the results of the *Sobel test* to corroborate whether there is effectively evidence of mediation. The fact that the observed *p*-value of the *Sobel test* does not fall below the established significance level of 0.05 indicates that the association between the independent variable of interest and the dependent variable is not reduced significantly by the inclusion of the mediator variable. We also estimate two additional models: one model that included all control and independent variables (Model 7) and one model that just included the independent variables (Model 8).

## Results

Table 1 provides the descriptive statistics (means and standard deviations) and the correlations of all the variables used in the empirical study. This table reveals that 38% of the schools have a female leader (i.e. the principal is a woman); the proportion of women on the management team is about 45%; and the proportion of women teachers is approximately 64%. The minimum and maximum values of the two latter variables are, respectively, 0 and 100%, and 40% and approximately 89%. These figures are well above the mean in most Spanish economic sectors and may be interpreted as evidence that the organisations considered in this study are largely female environments (Table 1).

**Table 1:** Means, standard deviations, variance inflation factors and correlations.

	Mean	s.d.	VIFs	1.	2.	3.
**1.** School students’ achievement	6.22	0.71				
**2.** Female principal	0.38	0.48	1.296	0.433**		
**3.** Women on management team	45.38	24.67	1.308	-0.006	0.453**	
**4.** Women teachers	64.18	9.87	1.396	0.466**	0.305**	0.244*
**5.** School ownership	0.59	0.49	1.882	-0.147	-0.025	-0.005
**6.** Foreign students	16.04	14.03	1.289	-0.493**	-0.024	-0.043
**7.** School size	41.76	18.81	1.540	0.233*	0.051	-0.094
**8.** Teacher stability	66.68	24.9	1.180	0.319**	0.104	0.113
**9.** Teacher motivation	31.12	18.01	1.332	0.388**	0.153	0.125
	**4.**	**5.**	**6.**	**7.**	**8.**	
**5.** School ownership	0.126					
**6.** Foreign students	-0.217*	0.132				
**7.** School size	0.104	0.608**	-0.088			
**8.** Teacher stability	0.154	-0.251*	0.058	-0.042		
**9.** Teacher motivation	0.368**	0.198*	-0.142	0.128	0.075	

***p<0.001

**p<0.01

*p<0.05

^†^p<0.10.

The third column in Table 1 shows that multicollinearity does not appear to be a problem in our study, since most of the explanatory variables (i.e. independent and control variables) have variance inflation factors (VIFs) that are well below the rule of thumb of 5 or 10 ― none exceeded 2 ― advocated by, respectively, Marquardt and Snee [140] and Kutner, Nachtsheim, and Neter [141].

Table 2 presents results from the hierarchical regression analysis performed.

**Table 2:** Hierarchical multiple regression analysis (dependent variable = *School students’ achievement*).

Variables	Model 1	Model 2	Model 3	Model 4
Constant	5.301*** (0.291) ^(1)^	5.165*** (0.279)	5.517*** (0.312)	4.272*** (0.457)
School ownership	-0.301^*†*^	-0.282^*†*^	-0.259	-0.373*
	(0.169)	(0.160)	(0.168)	(0.163)
Foreign students	-0.023***	-0.020**	-0.025***	-0.020**
	(0.006)	(0.006)	(0.006)	(0.006)
School size	0.015**	0.014**	0.013**	0.015**
	(0.004)	(0.004)	(0.004)	(0.004)
Teacher stability	0.005^*†*^	0.006*	0.006^*†*^	0.004
	(0.003)	(0.003)	(0.003)	(0.003)
Teacher motivation	0.013**	0.011**	0.015***	0.009*
	(0.004)	(0.004)	(0.004)	(0.004)
Female principal		0.389**		
		(0.124)		
Women on management team			-0.005^*†*^	
			(0.003)	
Women teachers				0.020**
				(0.007)
*R*^*2*^	*0*.*433*	*0*.*497*	*0*.*455*	*0*.*487*
*ΔR*^*2*^		*0*.065**	*0*.*023*^*†*^	*0*.054**
*Adjusted-R*^*2*^	*0*.*396*	*0*.*458*	*0*.*413*	*0*.*447*
*F-test*	*11*.746***	*12*.536***	*10*.596***	*12*.030***
*Durbin-Watson test*	*1*.*714*	*1*.*905*	*1*.*633*	*1*.*794*
	**Model 5**	**Model 6**	**Model 7**	**Model 8**
Constant	5.301*** (0.291)	4.298*** (0.437)	4.612*** (0.414)	4.482*** (0.399)
School ownership	-0.198	-0.347*	-0.263^*†*^	
	(0.152)	(0.157)	(0.147)	
Foreign students	-0.023***	-0.017**	-0.019**	
	(0.006)	(0.006)	(0.005)	
School size	0.015**	0.014**	0.010**	
	(0.004)	(0.004)	(0.004)	
Teacher stability	0.005^*†*^	0.005^*†*^	0.006*	
	(0.003)	(0.003)	(0.003)	
Teacher motivation	0.013**	0.007^*†*^	0.009*	
	(0.004)	(0.004)	(0.004)	
Female principal	0.542***	0.344**	0.499***	0.629***
	(0.125)	(0.122)	(0.120)	(0.138)
Women on management team	-0.009**		-0.009***	-0.008**
	(0.003)		(0.002)	(0.003)
Women teachers		0.017*	0.018**	0.029***
		(0.007)	(0.006)	(0.006)
*R*^*2*^	*0*.*565*	*0*.*537*	*0*.*608*	*0*.*377*
*ΔR*^*2*^	*0*.132***	*0*.104**	*0*.175***	
*Adjusted-R*^*2*^	*0*.*524*	*0*.*493*	*0*.*565*	*0*.*356*
*F-test*	*13*.909***	*12*.404***	*14*.334***	*17*.977***
*Durbin-Watson test*	*1*.*899*	*1*.*918*	*1*.*906*	*2*.*029*

^(1)^ Standard errors in brackets.

***p<0.001

**p<0.01

*p<0.05

^†^p<0.10.

Hypothesis 1 predicts that the presence of a woman in the position of principal will be positively related to school performance. The evidence provides statistical support for this hypothesis, as the coefficient of *female principal* is positive and significant (see Model 2 and Models 5–8, Table 2). Additionally, we performed a two-sample *t* test that revealed that there was a significant difference in *school students’ achievement* between secondary schools led by a woman and those by a man (*t* = 4.577, *p*<0.001). The average *school students’ achievement* for schools led by a woman was 0.63 points higher than the average achievement for schools led by a man.

Hypothesis 2a posits that a greater proportion of women on the management team will be positively related to school performance. The evidence shows that the relationship between *women on management team* and *school students’ achievement* is negative and significant (see Models 3, 5, 7, and 8, Table 2). These results are contrary to the arguments of our Hypothesis 2a, which is therefore rejected.

Hypothesis 2b held that *female principal* will mediate the relationship between the proportion of *women on management team* and school performance. First, a negative and significant relationship is found between *women on management team* and school performance in Model 3 (Table 2). Second, the logistic regression analysis revealed that the coefficient of *women on management team* has a positive and significant effect (Coefficient = 0.046; *p*<0.001; Cox and Snell *R*^*2*^ = 0.201, Nagelkerke *R*^*2*^ = 0.274) on the presumed mediator (i.e. *female principal*). Next, *school students’ achievement* is regressed on the control variables, *women on management team* and *female principal*. Model 5 shows that *female principal* is a significant predictor (*p*<0.001) of *school students’ achievement* when *women on management team* is also controlled (Table 2). In addition, Model 5 shows that the inclusion of the mediator in the model causes the coefficient for *women on management team* to increase (in absolute value), which indicates that the relationship between *women on management team* and *school students’ achievement* is not mediated by *female principal* (Table 2). This latter finding is contrary to one of the assumptions posited by Baron and Kenny that have to be fulfilled when testing mediating effects between variables [137]. Thus, these results do not provide statistical support for Hypothesis 2b. In order to corroborate the lack of such a mediation effect, we also conducted the *Sobel test*. The test statistic for the *Sobel test* was 0.323, with an associated *p*-value of 0.746. This indicates that the association between *women on management team* and school performance is not reduced significantly by the inclusion of the mediator in our model. In other words, there is no evidence of mediation of *female principal*.

Hypothesis 3a predicts that a greater proportion of women teachers will be positively related to school performance. The results provide statistical support for this hypothesis, as the coefficient of *women teachers* is positive and significant in all models in which this variable is considered (see Models 4 and 6–8, Table 2).

Hypothesis 3b suggested that *female principal* will mediate the relationship between a higher proportion of *women teachers* and *school students’ achievement*. A positive and significant relationship is found between *women teachers* and school performance in Model 4 (Table 2). The logistic regression analysis revealed that the coefficient of *women teachers* has a positive and significant effect (Coefficient = 0.069; *p*<0.01; Cox and Snell *R*^*2*^ = 0.091, Nagelkerke *R*^*2*^ = 0.124) on *female principal*. Model 6 also shows that *female principal* is a significant predictor (*p*<0.01) of *school students’ achievement* when *women teachers* is also controlled (Table 2). In addition, Model 6 shows that the inclusion of the mediating variable in the model causes the coefficient for *women teachers* to decrease, which ‘a priori’ could indicate that the relationship between *women teachers* and *school students’ achievement* might be partially mediated by *female principal* (Table 2). In this case, the test statistic for the *Sobel test* was 0.333, with an associated *p*-value of 0.739. Therefore, in light of these findings, we can conclude that there is no support for Hypothesis 3b either.

Finally, Table 2 reports that the five control variables considered are found to be significantly associated with the variable representing school performance in most models. Three of these variables (*school size*, *teacher stability*, and *teacher motivation*) have a positive and significant coefficient, while two other variables (*foreign students* and *school ownership*) have a negative and significant coefficient.

## Discussion and conclusions

In a time of passionate debate over the role women play within organisations, this study contributes to both theory and practice by exploring the relationship between the presence of women in different executive and non-executive positions and performance. Interestingly, the empirical evidence is obtained from a sample of service organisations that have been widely conceived as feminised environments—namely, secondary schools. We posit that because women are still underrepresented in leadership positions in most secondary schools, a positive relationship between schools led by a woman and with a greater proportion of women on the management teams and school performance is expected. On the other hand, given the specific context considered, we also assume a positive relationship between a greater proportion of women teachers and school performance. Finally, unlike most prior studies, we are also interested in testing the potential mediator role that can play a woman leader on the relationship between women on the management team and women teachers and school performance.

Overall, our results consistently show, in line with our expectations, that the relationship between women and school performance is positive if: 1) a woman occupies the highest level of the school hierarchy (*Hypothesis 1*) and 2) they make up a greater proportion of teachers (*Hypothesis 3a*). However, contrary to our expectations, our findings reveal the existence of a negative relationship between a high proportion of women on the management team and school performance (*Hypothesis 2a*). Also contrary to our expectations, our findings highlight the non-significant mediator role that a female principal has on the relationship between a greater proportion of women on the management team and also as teachers and school performance (*Hypotheses 2b* and *3b*, respectively). Therefore, our study finds that women may have a significant relationship with performance when they occupy a specific position in the school ladder but not when they simultaneously share different positions (in our case, as principal and on the management team, or as principal and teachers).

In light of the results, this study seems to confirm the benefits associated with women’s roles and values in terms of performance in secondary schools, as these benefits may positively influence students, but only in some specific situations. In this regard, note is taken of the usefulness and adjustment of a women leader to the needs of a complex service such as education, which often calls for many of the abilities and values attributed to the female gender according to *Social Role Theory*. These findings are also consistent with the results of other studies both involving educational organisations (for instance, schools) (e.g., [21]) and different samples of business firms (e.g., [37]).

Regarding the greater proportion of women on the management team, our study finds a negative relationship with school performance. At this point, it is interesting to note that the proportion of women on management team in our study sample is close to 50%. In some ways, our results suggest that if the proportion of women in this specific position further increased, it might be detrimental to school performance. This finding is contrary to some recent studies conducted in the business field that show that the greater the number of women on the board, the better the firm’s performance is (e.g., [142]). But it should be noted that in most these prior studies the proportion of women on boards/management teams is still very far below 50%.

It is true that our findings reveal that a female principal does not exert a significant mediator role in the relationship between a greater proportion of women on the management team and school performance. However, our results do not allow us to rule out the possibility that a greater number of female principals in the schools considered could have contributed to greater gender equality by creating more inclusive schools, at least in relation to the presence of more women on management teams. In fact, we also find that in schools with a female principal, the proportion of women in managerial positions is also greater than when the principal is a man. It can be seen that a female principal and women on the management team are also positively and significantly correlated. In this regard, our findings are consistent, at least to some extent, with some of the arguments from the *Similarity-Attraction Approach*, especially with those concerning the idea that women principals can offer more opportunities of sponsorship that advance women teachers’ careers toward managerial positions [86], [118]. Another difference is that then a positive and significant effect on school performance can be achieved. In any case, our findings also seem to be in consonance with several studies that have argued that high-status women have the most favourable attitudes toward other women as managers (e.g., [143], [144]). For example, Korabik and Abbondanza believe there is solidarity behaviour between women in management that leads to the formation of alliances, collaboration, shared aims, commitment to changing social structures for women, and expressions of loyalty and gender awareness in administrative practice [143].

Our findings also consistently reflect the fact that a greater proportion of women teachers is positively and significantly related to school performance. In this sense, given the specific nature of educational services, particularly secondary education, we emphasise that women teachers could manifest several values and attributes that are highly valued for meeting students’ needs at this educational level (e.g., [22], [24], [108], [109], [110]). Additionally, our results also revealed that schools with a proportion of women teachers above the average obtain better student achievement. Therefore, our findings also could justify, to some extent, the existence of a certain gender gap with respect to male teachers in secondary schools. From this standpoint, our results also seem to be in consonance with the *Similarity-Attraction Approach* that suggests the convenience of having a high proportion of women teachers, as it might encourage better communication, stronger cohesion, and lower conflict within schools [86]. However, contrary to the predictions drawn from the *Relational Demography Perspective* and, also to some extent, the *Similarity-Attraction Approach*, our results suggest a lack of a significant mediator role of a female principal on the relationship between a greater proportion of women teachers and school performance. Anyway, our findings do not preclude the possibility either that female teachers can feel more empowered when working in schools headed by female principals (for example, female principal and women as teachers are also positively and significantly correlated), which is also in line with prior research (e.g., [86], [88]), even though this has no significant effect on school performance.

### Implications for scholars and practitioners

This study has important implications for both scholars and practitioners. From a scholarly standpoint, the focus on female presence at different organisational levels may help advance our understanding of how a greater female representation operates even in a feminised environment. In this same vein, this study underscores the need to consider arguments from different theoretical approaches as an appropriate way to better understand the consequences (basically in terms of organisational performance) associated with a large female presence at different organisational levels. This study can also enable researchers to compare the main similarities and differences that may exist in terms of a greater female presence between more and less feminised organisational contexts. Moreover, this study raises some new issues, such as the extent to which it is actually positive, or not, that women are the dominant gender in most organisational positions.

In general, the results of this study confirm that women may play an important role in improving organisational performance in a feminised context not only when they represent the greater proportion of the workforce but also when they occupy the position of maximum responsibility within the organisation. Specifically, in the field of education, we champion the access of a greater number of women to positions of maximum responsibility, given the benefits this has for organisational performance. In this sense, we also suggest a professionalisation of management in Spain comparable to that of other European countries (e.g., [145], [146]). For example, France, the United Kingdom, Italy, Belgium, Germany, as well as most US states make numerous demands to access management and share similar modes of entry and competences. We believe that this policy would allow all women with such aspirations and suitable competences to reach that position. Women often face the need to prove additional competences and make more effort to reach the highest managerial position [116], [147]. Therefore, this policy may be fairer for women because it is not based on a choice informed by a chauvinist tradition in which more men occupy the highest position in the hierarchy. In this sense, and regardless of gender, the post of principal would be occupied by the candidate with the greater skills or competences for that position.

Another practical implication is closely related to the gender composition of the management teams. In this sense, we consider it might be fundamental to conduct a prior analysis of the context, needs, task type (strategic or operative) and task difficulty in each school, especially when there is already a certain gender balance. Based on this analysis, it may be easier to identify the roles and skills required, and thus also who can best play the required role regardless of gender, to improve management team performance and, ultimately, school performance.

A further practical implication arising from our findings has to do with the need for educational authorities to adopt measures to further increase the proportion of women teachers in secondary schools in Spain, at least in some regions. As noted above, overall, the proportion of women teachers at this educational level in Spain is lower than in most European countries. However, in our study sample the average proportion of women teachers in secondary schools is above the Spanish average, and this seems to have good consequences on school students’ achievement. In this regard, it is also interesting to note that students in the Community of Madrid obtain better academic results in relation to the Spanish average [148].

Finally, the results of this study also allow us to identify a series of implications associated with certain contextual and organisational factors that may improve an educational organisation’s performance. Regarding the contextual factors, the negative relation between the percentage of foreign students and school performance suggests an effort to improve the conditions in secondary schools where there are often more problems of adaptation, conflict, and school dropout. We believe that educational authorities should analyse each school more precisely in order to provide teachers and administrators in difficult contexts with the resources and training they need. With regard to teaching staff, our study demonstrates a positive relationship between school performance and increased teacher motivation. We therefore consider teachers’ recognition and incentive policies to be essential in a profession that offers limited opportunities for promotion in Spain.

### Limitations and future directions of research

The results of this study should be considered in the light of a number of limitations. First, limitations regarding the possible subjective nature of part of the information contained in the questionnaires, and therefore applicable in the construction of certain variables, would likewise be applicable here. While most of the measures of variables used in our study can be considered suitable (as most of them have also been used by other researchers), additional insights into their association may be gained by adopting measures of several variables of interest that reflect different perspectives. This could be the case, for example, for the variables associated with teacher motivation. It could be interesting to ask the teachers about their motivation to see whether they share the same perceptions.

Further research should also examine the mediating role of other organisational, contextual, or personal characteristics. This is undoubtedly a complex question that future research should address in order to gain a better understanding of the phenomenon under study. Although our performance measure is an objective and uniform measure for all schools considered in the study, it could also be interesting to use other alternative performance measures, such as winners of regional/national prizes, acquisition of desired skills and competences, and post-school performance. Additional research adopting a longitudinal design would also be necessary, as it might allow us to better identify the direction of the relationships (i.e. causality) between the main variables of interest. In this sense, for example, it could also be very interesting to examine how changes in gender composition over time in the different organisational positions considered in the study can affect later changes in performance and vice versa. Unfortunately, an analysis of this issue is beyond the scope of this study.

Likewise, the results obtained should be framed within the specific nature of the organisations analysed. Educational organisations (and, in the case of our study, secondary schools) can also be depicted as non-competitive environments or, in any case, less competitive environments than those of most business sectors. And women may feel, to a certain extent, more comfortable than men working in this type of environment. Niederle and Vesterlund, examining an environment where women and men perform equall y well, find that there are actually large gender differences in their propensity to choose competitive *vs*. non-competitive environments [78]. Therefore, in order to facilitate the generalizability of our findings, it would also be very interesting to examine in future research to what extent our results are also similar in other types of competitive and non-competitive sectors.

It could also be interesting to conduct a cross-industry study to control the potential effect of context. In any case, we trust our findings can be extrapolated not only to secondary schools but also to other educational organizations (such as, for example, primary schools, universities or business schools) and organisations in other sectors where women also represent the majority of the overall workforce, such as health or social work. Additional research is therefore needed to see to what extent this is true. Finally, future research could also benefit from using more secondary data. One of the main advantages of using this data source is the feasibility of both longitudinal and international comparative studies across similar or different types of organisations and countries (in terms of emerging, developing and developed countries), and also that they may be especially useful for making comparisons over time.

## Supporting information

S1 Questionnaire Information on the questions included in our questionnaire to carry out this paper. (PDF)

S1 Data Data set used in our empirical analysis. (SAV)
