# Supplementary material for: The female presence in different organisational positions and performance in secondary schools: Does a woman leader function as mediator?
Source: PLoS One. 2019 Sep 26;14(9):e0222411. doi: 10.1371/journal.pone.0222411 (PMC6762201; doi:10.1371/journal.pone.0222411)
Supplement: S1 Questionnaire — (PDF) [file pone.0222411.s001.pdf]

QUESTIONNAIRE

PUBLIC AND PRIVATE SECONDARY SCHOOLS

1. ISSUES CONCERNING THE SCHOOL AND ITS OPERATION

**1.1. School name**	

**1.2. School ownership**	□ **Public** □ **Private**

**1.4. Number of teachers in ESO and Bachillerato**	

**1.6. Socioeconomic and Cultural Level of the School Environment**			
□ **HIGH**	□ **MEDIUM-HIGH**	□ **MEDIUM-LOW**	□ **LOW**

**1.7. Are there students from other nationalities in ESO and Bachillerato?**<br>If your answer is yes, indicate that percentage.	□ YES ____%<br>□ NO

2. ISSUES CONCERNING THE MANAGEMENT TEAM

**2.1. Management Team Composition**<br>Mark with an X the number of people who make up the Management Team (principal, head teachers and school secretaries)	□ **1** □ **2** □ **3** □ **4** □ **5** □ **6 or more**

2.12. Principal information

**a) Gender**	□ **Male**	□ **Female**

2.13. Head Teachers information

2.13.1. Head Teacher in ESO

**a) Gender**	□ **Male**	□ **Female**

2.13.2. Head Teacher in Bachillerato

**a) Gender**	□ **Male**	□ **Female**

2.13.3. Other Head Teacher (specify _______________)

**a) Gender**	□ **Male**	□ **Female**

2.14. School Secretaries information

2.14.1. Secretary in ESO

**a) Gender**	□ **Male**	□ **Female**

2.14.2. Secretary in Bachillerato

**a) Gender**	□ **Male**	□ **Female**

2.14.3. Other Secretary (specify _______________)

**a) Gender**	□ **Male**	□ **Female**

3. ISSUES CONCERNING THE TEACHERS

**3.1. GENDER DISTRIBUTION**	**NUMBER OF MALE TEACHERS**	**NUMBER OF FEMALE TEACHERS**
	**___ Teachers**	**___ Teachers**

**3.3. DISTRIBUTION BY TENURE**	**% Teachers with less than 5 years**	**% Teachers with more than 5 years**	**Sum of boxes 100%**
	**___%**	**___%**	

3.12. GLOBAL ASSESSMENT OF TEACHERS ATTENDING THE PROFILE AND ROLE IN THE HIGH SCHOOL

Indicate, with respect to competence and attitude, the percentage of teachers over the total (100%) with the following characteristics:

Sum of boxes 100%

They have a high level of professional performance to achieve academic performance and show motivation, positive attitude and commitment to the school and the school community.	**___%**
They have a high level of professional performance to achieve academic performance but barely show commitment and cooperate little for the school development.	**___%**
They show involvement, willingness and commitment to the school and the school community but have few professional skills.	**___%**
They have few professional skills and hardly show commitment to the school, maintaining very low motivation.	**___%**
